# Hurricanes Irma and Maria and Diabetes incidence in Puerto Rico

**DOI:** 10.1186/s12889-023-15542-w

**Published:** 2023-05-30

**Authors:** Marijulie Martínez-Lozano, Carlamarie Noboa, Gerardo Alvarado-González, Kaumudi J. Joshipura

**Affiliations:** 1 Center for Clinical Research and Health Promotion, University of Puerto Rico, Medical Sciences Campus Suite A 107, Box 365067, San Juan, Puerto Rico, 00935 USA; 2 Corporación de Servicios de Salud y Medicina Avanzada (COSSMA), Puerto Rico, USA; 3 School of Public Health, Ahmedabad University, Ahmedabad , India; 4 Department of Epidemiology, HARVARD SCHOOL OF PUBLIC HEALTH, Boston , USA

**Keywords:** Type 2 diabetes, Hurricane, Health care, Natural disasters, Puerto Rico

## Abstract

**Objective:**

To evaluate the impact of Hurricanes Irma/Maria on diabetes incidence in Puerto Rico. Mortality increased substantially after the hurricanes, but morbidity was not assessed.

**Methods:**

We recruited 364 participants from the San Juan Overweight Adults Longitudinal Study (SOALS) aged 40–65 years who completed a three-year follow-up and were free of diabetes. We conducted additional questionnaires 1.7–2.5 years after hurricanes. Glycosylated hemoglobin (HbA1c), fasting glucose and insulin were assessed at all three visits. We compared diabetes incidence between pre-hurricane visits and between visits spanning the hurricanes using Generalized Estimating Equation (GEE) adjusting for within person repeated measures, age, and body mass index (BMI).

**Results:**

Diabetes incidence was significantly higher spanning the hurricanes than pre-hurricane (multivariate GEE model: IRR = 2.1; 95% CI: 1.4–3.1). There was a significantly higher increase spanning the hurricanes compared to pre-hurricanes for Homeostatic Model Assessment of Insulin Resistance (HOMA-IR) (median: 0.3 uIU/mL vs. 0.2 uIU/mL). HbA1c levels increased by 0.4% spanning the hurricanes.

**Conclusion:**

Increases in diabetes incidence, HOMA-IR and HbA1c were higher spanning the hurricanes compared to the pre-hurricanes period. The increase in diabetes incidence remains significant after adjusting for age and BMI.

## Introduction

Prior studies suggest that hurricanes could lead to increased diabetes incidence and related complications, hospitalization, and mortality [1–7]. After natural disasters, anxiety, overeating, interruption of medications, and disruption of water supply and access to nutritious food could lead to glucose abnormalities; this in turn could lead to increased risk for diabetes and related comorbidities or complications and hospitalizations [1–3]. People with diabetes need to follow medical treatment and diet recommendations to manage their diabetes and keep their glucose and glycosylated hemoglobin (HbA1c) levels within an acceptable range. This might be more challenging after natural disasters. Also, insulin may be difficult to obtain after hurricanes, and difficult to store adequately during power outages, which could lead to poor control of diabetes.

In the week after Hurricane Sandy, the rate of diabetes diagnosis in the emergency room visits in New Jersey increased by 84% [1]. Patients with diabetes had more serious complications after hurricanes due to disrupted health care services and limited resources [4]. Moreover, the annual rate of death from diabetes in the 12 months following Hurricane Iniki in Kauai in 1992, was 2.6 (95% CI: 1.4–4.7) times higher than the median annual rate in 1987–1991 [5]. After the 2004 hurricane season in Florida, 5% of the indirect mortality from storm-related deaths were from diabetes [6].

Several studies have identified barriers that prevent people from preparing adequately for a natural disaster [8–11]. In 2012, a review suggested that only 30-40% of United States (US) residents had emergency supplies at any given time [9]. Low preparedness is common even in areas that have previously experienced natural disasters, such as hurricanes [10]. Exposure to natural disasters is a consistent predictor of individual adverse health outcomes, including the development or worsening of chronic diseases [12, 13]. It seems likely that high risk individuals without diabetes could experience impaired glucose metabolism, elevated HbA1c levels and increased risk for diabetes.

Puerto Rico (PR) is located in a hurricane prone area in the Caribbean and Atlantic basin. In September 2017, Hurricanes Irma and Maria impacted Puerto Rico leaving a large part of the population without power, water, and/or telecommunications for over six months in some areas, with disruption of primary health care services, and food supply. By December 2017, the PR government reported 64 attributed deaths to Hurricane Maria. However, in 2018 a study from George Washington University estimated 2,975 deaths, which was later accepted as the official death toll [14]. Diabetes is the third highest cause of death in Puerto Rico [15]. One recent study in the US Virgin Islands, found that the high rates of diabetes related morbidities after Hurricanes Irma and Maria were perceived to be due to the lack of availability of insulin [16]. The prevalence of diabetes in Puerto Rico was 15.5% (95% CI: 14.3-16.6%) in 2018 [17], compared to 10.5% in the general US population [18]. People who are less prepared may be more likely to have a health impact due to hurricanes [19], and may likely have increased risk for diabetes.

Despite the high burden of hurricanes and diabetes in Puerto Rico, the impact of hurricanes on diabetes incidence has not been assessed. US studies relating natural disasters and diabetes mostly focused on people with diabetes and did not evaluate diabetes incidence. This study prospectively evaluates the impact of Hurricanes Irma and Maria on diabetes incidence and management in Puerto Rico.

## Research design and methods

### Study population

The Preparedness to Reduce Exposures and diseases Post-hurricanes and Augment Resilience (PREPARE) study was launched to longitudinally evaluate the impact of Hurricanes Irma and Maria on health in Puerto Rico, and to evaluate hurricane preparedness in this context. Diabetes was of key interest in PREPARE; the study design, population and methods are described in our prior publication [19].

PREPARE study was conducted among participants who were enrolled in a previous cohort study named San Juan Overweight Adults Longitudinal Study (SOALS). SOALS had pertinent pre-hurricane data from assessments conducted at baseline (2011 to 2013) and at three-year follow-up visits (2014 to 2016). SOALS recruited overweight/obese Hispanic adults aged 40 to 65 years, without diabetes mellitus (DM) and major conditions including cardiovascular disease. At baseline, participants reporting physician-diagnosed type 1 or type 2 diabetes, taking insulin or oral anti-hyperglycemic agents, or classified as having diabetes based on study assessments were excluded. At SOALS follow-up exam, participants reporting a diagnosis of diabetes, were given a medical referral and advice to consult their health care provider to seek a definitive diagnosis. We requested participants to send us a copy of their laboratory test results. If the diabetes was not confirmed, we invited the participant to undergo the oral glucose tolerance test and glucose assessments and classified their diabetes status based on study measures.

Participants who were considered free of diabetes at the follow-up visit and had previously consented to be contacted for future studies were potentially eligible for PREPARE study (N = 869). Since SOALS consisted of adults drawn primarily from San Juan and neighboring areas, and since the hurricane impact varied by location, we stratified the sampling by location to get better representation across Puerto Rico. We aimed to recruit 125 SOALS participants from three locations: the capital city (San Juan), the rest of the San Juan Metropolitan area (including Bayamón, Carolina, Cataño, Guaynabo and Trujillo Alto) and from municipalities of Puerto Rico outside of San Juan Metropolitan Area.

### Procedures

Recruitment and post-hurricane data collection for PREPARE were conducted in a health clinic of the University between May 2019 and July 2020, 17–34 months after the hurricanes. After completing written informed consent, and confirmation of eligibility, trained interviewers conducted computer-aided interviews in Spanish using REDCap. We also assessed blood pressure, blood glucose and HbA1c levels, and anthropometric measures. Hence, we could compare measures across 3 timepoints (SOALS baseline, SOALS follow-up and PREPARE visit) and two time periods: (1) baseline to follow-up (pre-hurricanes: follow-up minus baseline; median 3.0 years) and (2) follow-up to PREPARE visits (spanning hurricanes: PREPARE minus follow-up; median 4.7 years).

The study was impacted due to a series of earthquakes that impacted Puerto Rico in January 2020, and the government mandated lockdown starting March 15, 2020 due to the COVID-19 pandemic. Due to this we decided to drop the visits for the 11 pending participants, hence, PREPARE study included a total of 364 participants.

### Diabetes assessments in SOALS and PREPARE

For all visits, participants were asked to fast for 10 hours prior to their appointments, and through the last blood draw. For SOALS baseline and follow-up, blood samples were drawn at fasting, and after a 75 g oral glucose tolerance test (OGTT) at 30-mins, 1 and 2 hours. Only fasting blood was drawn in PREPARE for assessment of glucose, insulin and HbA1C. To compare diabetes prevalence and incidence measures, since OGTT was only assessed pre hurricanes, we used the fasting glucose and HbA1c measures to classify diabetes across all time points and time periods. We considered participants who had study assessed fasting glucose ≥ 126 mg/dl, or HbA1c > 6.5% (48 mmol/mol) as having diabetes. People were classified as having pre-diabetes if they had fasting glucose 100–126 mg/dl or HbA1c 5.7–6.5% (39–48 mmol/mol), or as having normal glycemia if all these values were below the mentioned thresholds for pre-diabetes [20]. Homeostatic Model Assessment of Insulin Resistance (HOMA-IR) was calculated using fasting values as [glucose (nmol/L) * insulin (µU/mL)/22.5] [21].

### Statistical methods

People with missing data for key variables were excluded or dropped from the pertinent analyses. Descriptive analyses were performed using PREPARE data. To assess the pre-hurricanes diabetes incidence rate (IR), we included 968 eligible participants without diabetes at baseline who completed the SOALS follow-up. For the incidence rate spanning the hurricanes, three participants who reported diabetes in the post-hurricanes visit but did not have study measures consistent with diabetes were excluded from this analysis. Since we excluded people who developed diabetes prior to each time point for incidence measures, the participants varied across the two time-points. Hence we computed adjusted incidence rate ratio (IRR) using a generalized estimating equation (GEE) model adjusting for within person repeated measures and key potential time varying confounders, age and body mass index (BMI) [22]. We also evaluated crude incidence rate within subgroups of income and education using survival analysis to see any differences in diabetes development across these subgroups.

Changes in fasting glucose, HOMA-IR and HbA1c were computed in the same 364 people for the two time periods. Normality of the changes were evaluated using Shapiro-Wilk test. Since the evaluated measures were not normally distributed, Wilcoxon signed-rank tests were conducted to compare changes in these measures across the two time periods. Since the effect of the hurricanes on HOMA-IR and HbA1c may vary across gender, education, income, and location, we used Wilcoxon rank-sum test to evaluate whether changes in HOMA-IR, and HbA1c levels spanning the hurricanes were different across subgroups defined by these factors. A significance level of ≤ 0.05 was used in all analyses. We used Stata software version 16.1 to conduct statistical analyses, and R-Studio for the figures.

## Results

The mean age at the time of PREPARE visit was 58.7 years (SD = 6.8) and 76.9% were women (Table 1). Over half of the participants (51.9%) reported a family income of $20,000 or more, while 73.9% had higher than high school education; 42.0% were employed and 39.3% were retired/disabled. Almost half (47.8%) the participants were classified as having pre-diabetes prior to the hurricanes, and 66.4% at the time of the post-hurricane visit.

**Table 1:** Sociodemographic and hurricane impact variables (at the time of the PREPARE visit)

	(N = 364)
	N (%) | M (SD)
**Age**	58.7 (SD = 6.8)
**Gender**	
Female	280 (76.9%)
Male	84 (23.1%)
**Income**	
Less than $20,000	175 (48.1%)
$20,000 or more	189 (51.9%)
**Education**	
High School or less	95 (26.1%)
More than High School	269 (73.9%)
**Employment status**	
Employed	153 (42.0%)
Unemployed	29 (8.0%)
Retired/ Disabled	143 (39.3%)
Homemaker	39 (10.7%)
**Location**	
San Juan	144 (39.6%)
Metropolitan	116 (31.9%)
Outside San Juan Metropolitan Area	104 (28.6%)
Diabetes status before hurricanes*	
No diabetes (HbA1c < 5.7%)	190 (52.2%)
Pre-diabetes (HbA1c 5.7-6.4%)	174 (47.8%)
Diabetes (HbA1c ≥6.5%)	-
Diabetes status after hurricanes**	
No diabetes (HbA1c < 5.7%)	74 (20.4%)
Pre-diabetes (HbA1c 5.7-6.4%)	241 (66.4%)
Diabetes (HbA1c ≥6.5%)	48 (13.2%)

*SOALS follow-up visit

**One participant did not have HbA1c measurements but reported being diagnosed with diabetes after the hurricanes, therefore, total is 363.

N = Number of participants; M = mean; SD = standard deviation.

Between the pre-hurricanes visits, there were 61 new diabetes cases (IR = 2.1 per 100 person-years; 95% CI: 1.7, 2.8), compared to 44 new cases spanning the hurricanes (IR = 6.7 per 100 person-years; 95% CI: 5.0–9.0), which translates to IRR of 3.1 per 100 person-years; 95% CI: 2.1–4.7 (Table 2). Incidence rate ratio (IRR) comparing the incidence rates before and spanning the hurricanes adjusted for within person repeated measures using GEE was 2.0 (95% CI: 1.4–2.8), and additionally adjusting for age and BMI was 2.1 (95% CI: 1.4–3.1).

**Table 2:** Diabetes incidence rates (IR) and incidence rate ratios (IRR)

	Pre-hurricanes Rate N = 968 (95% CI)	Spanning hurricanes Rate N = 360 (95% CI)	Comparison (95% CI)
**IR**	2.15 (1.67, 2.76)	6.71 (4.99, 9.02)	--
**Crude IRR**			3.12 (2.07, 4.68)
IRR adjusted for within person repeated measures* (N = 968)			1.99 (1.40, 2.83)
IRR adjusted for BMI and age* **(N = 952)**			2.12 (1.45, 3.11)

*Using GEE model.

Diabetes was defined as having an HbA1c ≥ 6.5%.

Time period: pre-hurricanes (2014–2016) and spanning the hurricanes (2019–2020).

CI = confidence interval.

Participants reporting annual family income below $20,000 had higher incidence spanning the hurricanes (IR = 7.6 per 100 person-years; 95% CI: 5.1–11.3) compared to those with higher income (IR = 5.9 per 100 person-years; 95% CI: 3.8–9.1) (Table 3). Similarly, participants with high school or less education (IR = 7.4 per 100 person-years; 95% CI: 4.3–12.7) had higher incidence spanning the hurricanes compared to those with higher education (IR = 6.5 per 100 person-years; 95% CI: 4.5–9.2). Participants with a family income below $20,000 had an IRR of 4.2 (95% CI: 2.3–7.8) comparing the incidence rates before and spanning the hurricanes, while participants with higher income had lower IRR 2.4 (95% CI: 1.3–4.2). For participants with high school or less education, IRR comparing the incidence rates before and spanning the hurricanes was also higher (IRR = 3.5; 95% CI: 1.8–6.6), than for participants with higher education (IRR = 2.4; 95% CI: 1.1–6.1).

**Table 3:** Diabetes incidence rates (IR) and incidence rate ratios (IRR) by income and education

	Pre-hurricanes Incidence Rate N = 968 (95% CI)	Spanning hurricanes Incidence Rate N = 360 (95% CI)	Crude IRR (95% CI)
**Income**			
Less than $20,000	1.8 (1.2–2.7)	7.6 (5.1–11.3)	4.2 (2.3–7.8)
$20,000 or more	2.4 (1.8–3.4)	5.9 (3.8–9.1)	2.4 (1.3–4.2)
**Education**			
High School or less	2.1 (1.6–2.7)	7.4 (4.3–12.7)	3.5 (1.8–6.6)
More than High School	2.6 (1.3–5.3)	6.5 (4.5–9.2)	2.4 (1.1–6.1)

CI = confidence interval

Diabetes was defined as having an HbA1c ≥ 6.5%.

Time period: pre-hurricanes (2014–2016) and spanning the hurricanes (2019–2020).

**Fig. 1:**
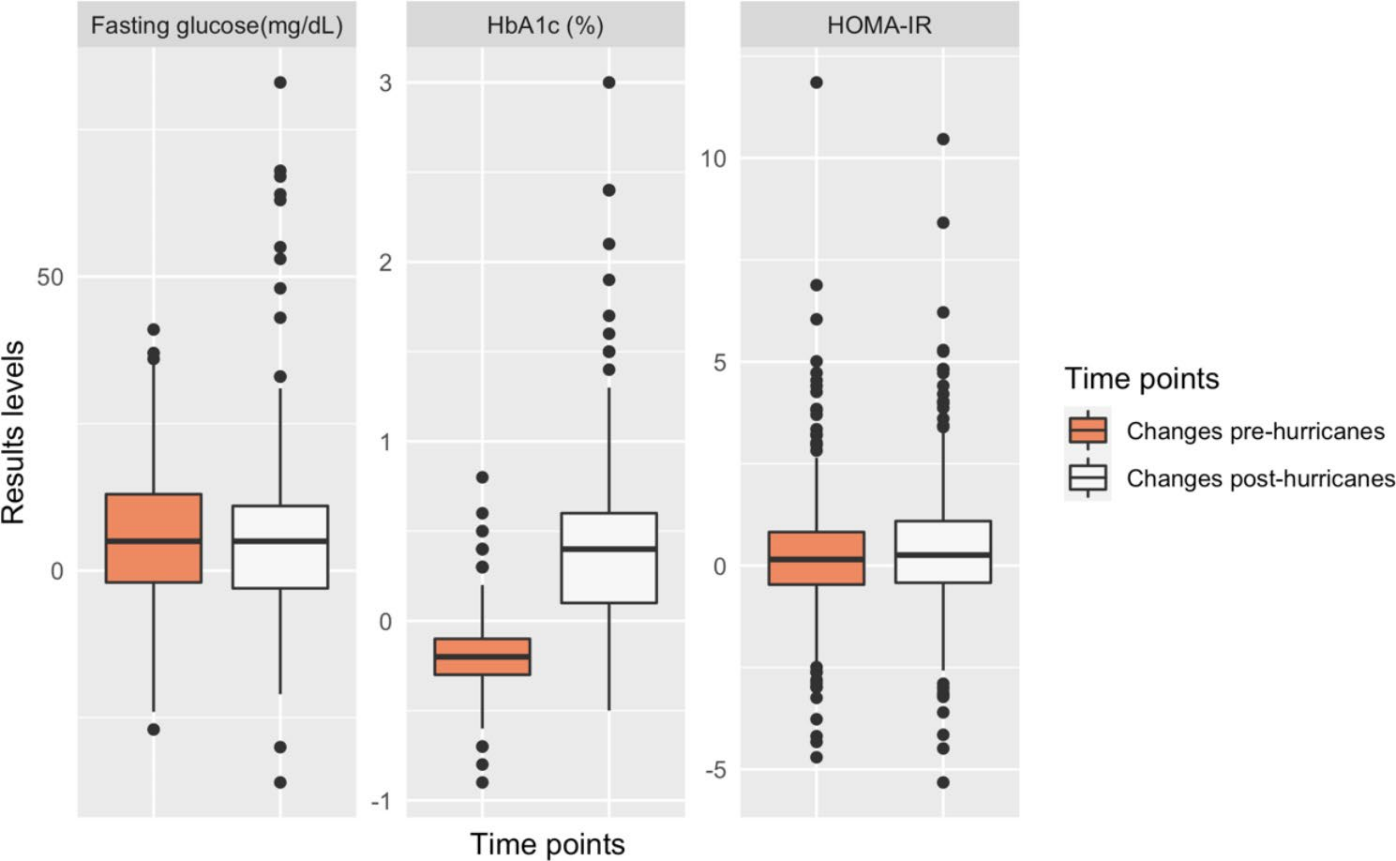
Changes in blood sugar level before and after the hurricanes Time points: pre (2014–2016) and post-hurricanes (2019–2020).

There was a significant increase in the changes between pre-hurricanes visits to changes between visits spanning the hurricanes for HOMA-IR (p = 0.05) and HbA1c levels (p < 0.001) (Fig. 1). HOMA-IR levels increased more spanning the hurricanes (median 0.3 uIU/mL), compared to pre-hurricane (0.2 uIU/mL). HbA1c levels decreased by 0.2% (median) between the pre-hurricane visits and increased by 0.4% spanning the hurricanes. Changes in fasting glucose levels remained similar (5.0 mg/dL) across the two time periods. Females had a significantly higher increase in HbA1c (median increase = 0.4%) spanning the hurricanes compared to males (median increase = 0.3%). Similarly, HOMA-IR levels increased significantly more in females (median increase = 0.3) than in males (median increase = 0.03). HOMA-IR and HbA1c changes spanning the hurricanes were similar across subgroups defined by education, location, and income (not shown in tables).

## Discussion

Prior studies had documented the impact of natural disasters on patient with diabetes. A recent study after Hurricanes Irma and Maria in the US Virgin Islands [16] reported that physicians and medical record staff perceived higher rates of diabetes-related morbidities. The same study also found a significant increase in the percentage of patients’ visits for endocrine, nutritional, and metabolic diseases; more than half of the visits were attributable to diabetes or related illnesses. Previous studies have found that people with diabetes needed support in the week after disasters due to increased risk of myocardial infarction [23]. In the aftermath of Hurricane Katrina, one of the main concerns for people with diabetes was obtaining insulin/medication and appropriate care for their condition. However, the impact of natural disasters on diabetes incidence is an understudy topic. According to the Behavioral Risk Factor Surveillance System (BRFSS), the prevalence of diabetes in Puerto Rico, for 2019, was 16.7% (95% CI: 15.6-17.8%), which is 1.2% higher than in 2018 [17]. To our knowledge, this is the first study to report a significant increase in diabetes incidence after a natural disaster and to highlight diabetes incidence after natural disasters in PR - a US territory prone to hurricanes with a majority Latino population and high federal poverty rate.

Changes in fasting glucose and HOMA-IR spanning the hurricanes were significantly higher than changes between the pre-hurricane visits. HbA1c increased more spanning hurricanes compared to earlier. The average time between the pre-hurricane visits was 3.0 years (SD = 0.25), whereas it was 4.7 years (SD = 0.46) between the visit spanning the hurricanes. However, the difference in the incidence rates cannot be attributed to the difference in the two time periods since the incidence rate ratio considers the time in the denominator. Considering that the higher incidence persisted after adjusting for age and BMI, the incidence may likely have increased due to the impact of the hurricanes. This is supported by the fact that participants with less income and education had higher increase in diabetes incidence spanning the hurricanes despite having lower pre-hurricanes incidence compared to participants with higher income and education. Since we compared glucose abnormalities across time points within the same individuals, results from these comparisons are also unlikely to be confounded. Our data is not representative of the whole Puerto Rican population which may affect generalizability. However, the associations are still likely to be valid.

Literature has usually focused on natural disasters and the impact on patients with diabetes [4, 6, 7]. However, given our findings of increase in diabetes incidence after the hurricanes, it is important to consider that high-risk people may likely further increase their risk of developing diabetes after hurricanes due to a combination of factors such as lack of water, power, nutritious foods, physical and mental stress. Tanaka and colleagues found that the high amount of stress after a hurricane activates the body’s “fight-or-flight” system for longer periods of time, which can lead to the development of diabetes [24]. Social determinants of health like income and education can also have an effect in the population’s health, as after natural disasters low-income people tend to have a higher impact. Our results showed that the incidence rate of participants with lower income and educational attainment increased significantly after the hurricanes demonstrating that social economic disparities resulted in higher risk for developing diabetes.

Detrimental changes in diet due to hurricanes could also have contributed to increased risk for diabetes. In the aftermath of the hurricanes in Puerto Rico, most (41%) of the food received at a federal distribution center were snacks and sweets [25]. Only 13% of the food was fruits, 4% vegetables, 13% proteins and 7% grains; 46% of these foods were high in sodium, saturated fat or added sugar [25]. In PREPARE, long-term diet changes were similar among people who had diabetes, pre-diabetes or normoglycemia before the hurricanes. No associations were found between long-term diet changes post-hurricanes and the changes in HbA1c spanning the hurricanes. In one study, HbA1c and fasting insulin levels rose significantly post-hurricanes [7]; however, we did not find any studies evaluating HOMA-IR or fasting glucose after hurricanes.

Our results suggest that the incidence of diabetes may have increased in Puerto Rico following Hurricanes Irma and Maria. The results were adjusted for age, suggesting that the increase in diabetes incidence spanning the hurricanes was beyond the expected increase in diabetes incidence due to aging. These findings have high public health relevance, as high-risk adults, those with lower socio-economic resources and people with pre-diabetes should take additional preparedness measures for natural disasters to sustain healthy levels of HbA1c. After a natural disaster the medical attention is often concentrated in treating acute injury and illness, leaving fewer resources to prioritize chronic conditions prevention and management. In 2007, the Disaster Response Task Force from the American Diabetes Association released a statement on Emergency and Disaster Preparedness, which contained a guide for people with diabetes [26]. A similar guide is needed for patients at high-risk of diabetes, which can incorporate recommendations such as storing nutritious food options that have a long shelf-life, staying well hydrated, and stress management skills to prevent the development of diabetes and its complications. The relevance of these findings also can extend globally and add to existing evidence that natural disasters could have a detrimental impact on the population’s health and increase social disparities. Additional research and global efforts are needed to further understand the impact of diabetes incidence after natural disaster.

## Data Availability

The datasets generated and/or analyzed during the current study are not publicly available as the grant just ended in 2021 and the primary manuscripts are still in progress, but the data are available from the corresponding author on reasonable request.

## Abbreviations

BMI: Body mass index
BRFSS: Behavioral Risk Factor Surveillance System
GEE: Generalized Estimating Equation
HbA1c: Glycosylated hemoglobin
HOMA-IR: Homeostatic Model Assessment of Insulin Resistance
IR: Incidence Rate
IRR: Incidence Rate Ratio
PR: Puerto Rico
PREPARE: Preparedness to Reduce Exposures and diseases Post-hurricanes and Augment Resilience
OGTT: Oral glucose tolerance test
SOALS: San Juan Overweight Adults Longitudinal Study
CI: Confidence interval
